# The Cost Effectiveness of Hand Held Ultrasound Scanning for Abdominal Aortic Aneurysm in Older Males with a History of Smoking

**DOI:** 10.36469/9856

**Published:** 2013-07-26

**Authors:** Denver Phiri, Peter J. Mallow, John A. Rizzo

**Affiliations:** 1 GE Healthcare, Chalfont St Giles, UK; 2 S2 Statistical Solutions, Inc., Cincinnati, OH USA; 3 Stony Brook University, Stony Brook, NY USA

**Keywords:** cost effectiveness, abdominal aortic aneurysm, hand held ultrasound, simulation model, cost utility analysis, economic evaluation

## Abstract

**Objective:** Abdominal aortic aneurysm (AAA) is a serious illness occurring in 1 of 20 older men. Guidelines emphasize the role of ultrasound scanning for patients at risk of AAA, yet the cost effectiveness of such scanning remains uncertain. New pocket mobile echocardiography (PME) devices may enhance the cost effectiveness of such scanning due to its low cost, ability to be used in primary care settings, and high degree of accuracy. This study performs cost utility analyses (CUAs) comparing opportunistic scanning for AAA using a PME to usual care for a hypothetical cohort of 10,000 male smokers age 65+.

**Methods:** The study compares the incremental cost per quality-adjusted life year (QALY) gained for three alternative strategies over a 5-year time horizon. The study used a decision analytic simulation model to calculate the incremental cost utility for the different strategies. Three alternative criteria for surgical intervention were considered via scanning according to aneurysm size. These treatment strategies were compared to a control group that received no scanning. Model input values are taken from the literature. Sensitivity analysis was performed to gauge the robustness of the results.

**Results:** Opportunistic scanning is cost effective. Indeed, when surgical intervention is limited to medium (5.0-5.4 cm) or large (≥5.5 cm) aneurysms, such scanning is dominant; that is, it costs less and increases QALYs compared to usual care. When surgical intervention is extended to small (4.0-4.9 cm) aneurysms, scanning remains cost effective ($64,156 per QALY vs. $100,000 threshold). The results are robust to alternative plausible model input values.

**Conclusion:** These findings suggest that primary care physicians with proper training should consider PMEs as a cost effective method to opportunistically scan and manage AAA patients among older males who have a history of smoking.

## BACKGROUND

Abdominal aortic aneurysm (AAA) is a serious illness occurring in 1 of 20 older men and is the 15th leading cause of death in the United States due to the complications of an aortic rupture.[1] AAA is a chronic, progressive disease that often requires surgical repair. The risk of rupture and death both increase with aneurysm size. This raises the question as to what aneurysm size is optimal for surgical intervention. Current practice favors intervention once the AAA has reached 5.5 cm.[2] Scanning patients to identify the presence of an aneurysm and monitoring its growth is an important diagnostic practice for managing these patients.

Clinical examination for AAA detection using abdominal palpation remains inaccurate.[3] Recent guidelines emphasize the role of systematic ultrasound scanning for patients at risk of AAA.[4] Ultrasonography is the preferred method of scanning because of its accuracy, patient acceptance, lack of radiation exposure, and wide availability.[5] The sensitivity and specificity of ultrasonography for AAA identification and assessment are nearly 100%, with inaccuracies usually resulting from minor measurement variations rather than failure to distinguish between large aneurysms and normal aortas.[5] However, diagnostic imaging modalities such as traditional ultrasound scans are typically reserved for patients with known aneurysms or risk factors such as family history of AAA or heart disease.[4] These scans also require follow-up visits by the patient to a specialized facility that has the ultrasound equipment. Despite recent legislation – The Screening Abdominal Aortic Aneurysms Very Efficiently Act (SAAAVE) - paying for the cost of ultrasound screening for new Medicare enrollees who have known risk factors for AAA, the number of people receiving the ultrasound exam did not increase as envisioned in the legislation.[6] As a result, many patients aged 65 and above may have undetected AAA and be at risk for rupture, and death.

A less costly and more convenient method for assessing the presence of AAA in a large group of patients aged 65 and above may help to identify and treat aneurysms early, thus avoiding costly emergency treatment and death. Recent advances in ultrasound technology include hand-held, pocket-sized devices that may greatly enhance the feasibility of in-office scanning due to their convenience and low cost.[7] These pocket mobile echocardiography devices (PMEs) have been shown to yield accurate assessments[1] and provide the same level of diagnostic accuracy in AAA scanning during physical examinations as a conventional echographic evaluation.[8] Using these devices, a "quick scan" approach requiring less than 5 minutes has been found to have a high level of effectiveness.[8] This enables the primary care physician to perform in-office scanning and surveillance for AAA without the need for additional health care staff and associated costs, or the need for further outpatient testing. The patient benefits from reduced waiting times and not having to schedule a separate visit for the ultrasound.

While the effectiveness and accuracy of portable echoscopes has been recognized, evidence on the cost effectiveness of this new technology is lacking. To bridge this gap in the literature, the present study investigates whether the use of this hand held device is cost effective in a hypothetical population of 10,000 older (e.g., age 65 years and above) male subjects with a history of smoking who would likely not receive a scan.[6] One-time scanning for AAA is currently recommended for male smokers age 65 to 75,[9] as smoking places them at higher risk of AAA.[10]

## METHODS

Cost utility analyses (CUAs) were performed comparing opportunistic scanning for AAA using a PME device to usual care in a hypothetical cohort of 65+ year-old male smokers who otherwise had no risks for AAA that would warrant scanning via ultrasound. A simulation model was developed comparing the incremental cost per quality-adjusted life year (QALY) gained for three PME scanning cohorts (Scenarios II – IV) to a usual care cohort (Scenario I), defined as no scanning (Figures 1 & 2). The analysis was conducted from the third-party payer perspective and considered a 5-year time horizon. Model input values were derived from the literature. The simulation model was developed using Microsoft Excel.

**Figure 1.** Decision Model Scenario I

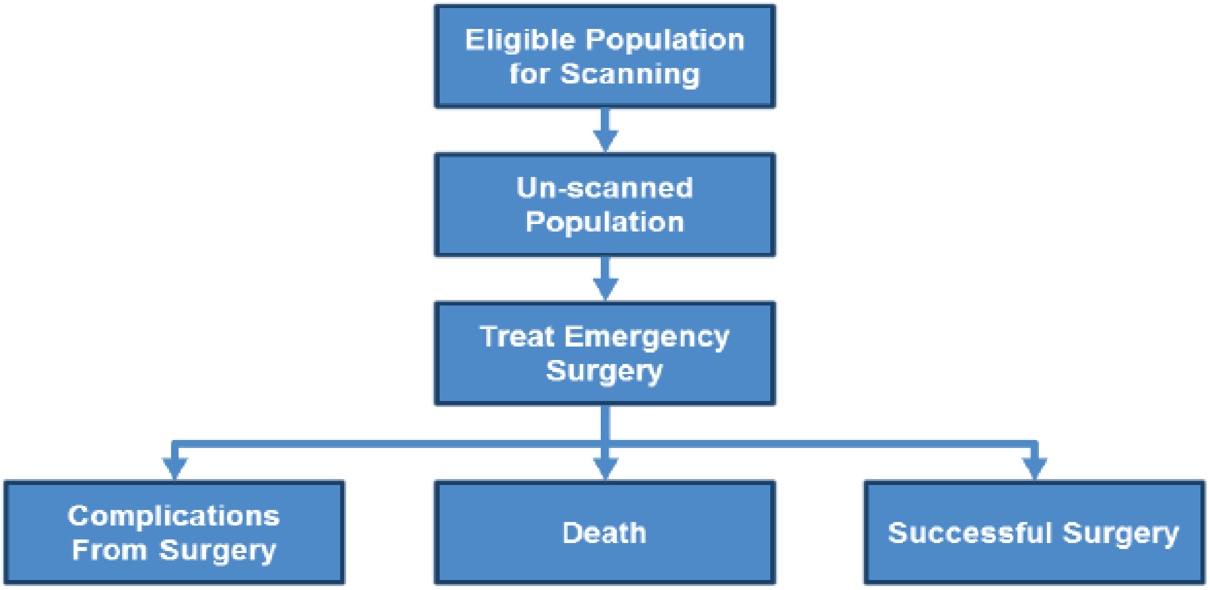

**Figure 2.** Decision Model Scenario II-IV

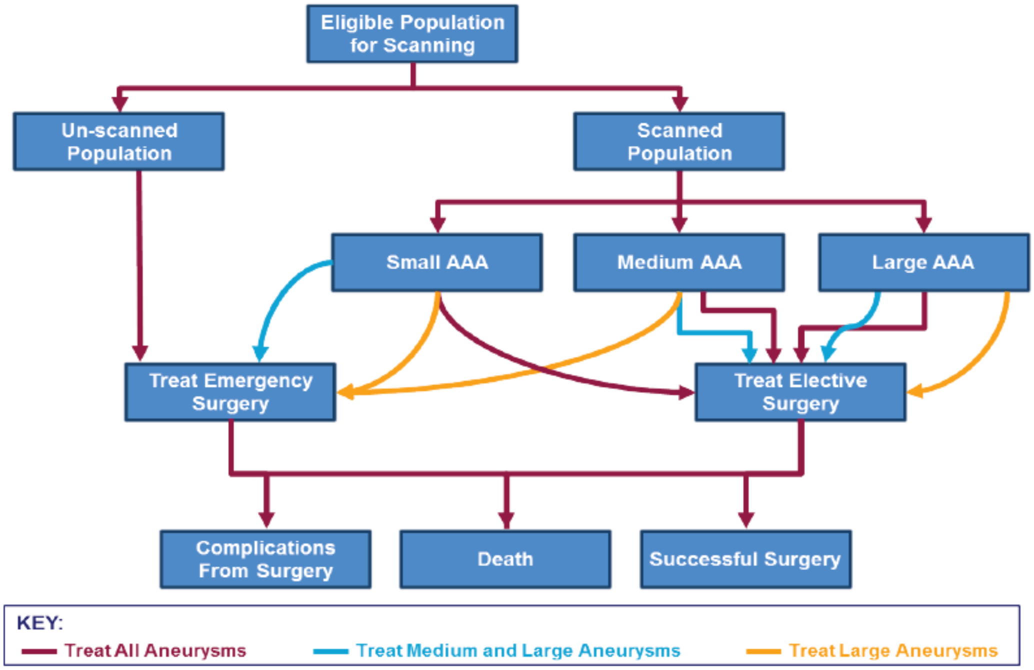

**Study Question**

Early detection of aneurysms allows the opportunity for a safer and less costly surgical intervention. The cost effectiveness question then becomes: are the cost savings from less risky surgeries and improvements in life expectancy sufficient with scanning to justify the additional costs of scanning? The answer to this question depends in part on how patients whose aneurysms have been identified via scanning are subsequently treated. These aneurysms may be categorized into three groups: small (e.g., 4-4.9 cm), medium (e.g., 5-5.4 cm) and large (e.g. ≥5.5 cm). Small aneurysms typically grow more slowly and are less likely to rupture. Hence, operating on small aneurysms is much less likely to avert rupture and death than operating on larger ones. In other words, greater expected cost savings and improvements in life expectancy should be had from operating on larger rather than smaller AAAs, other things being equal.

**Model Cohorts**

The simulation model quantified the expected costs and expected benefits in four cohorts of 10,000 patients each, a usual care cohort, and three scanning cohorts:

> **Scenario I:** Usual care, no scanning: Because it was assumed that no patients were scanned in the usual care arm, aneurysms become apparent only when they rupture and must be treated on an emergency basis (Figure 1).

Untreated aneurysms are assumed to grow over time at rates that vary depending on the initial size of the aneurysm; these rates are taken from the literature. Aneurysm growth increases the risk of rupture. This analysis considers three clinical strategies for surgical treatment of patients whose aneurysms have been detected via scanning. The scenarios differ in terms of the size at which aneurysms are treated surgically:

> **Scenario II:** Surgically treat any patient with a small (4.0 to 4.9 cm), medium (5.0 to 5.4 cm) or large (≥ 5.5 cm) aneurysm.
>
> **Scenario III:** Surgically treat any patient with a medium or large aneurysm.
>
> **Scenario IV:** Surgically treat only those patients with a large aneurysm (Figure 2).

This analysis also compared the cost effectiveness of scanning and treating all identified aneurysms (Scenario II) to Scenarios III and IV, respectively, which only treated a portion of aneurysms.

In the scanning arms, an assumption was made that 100% of patients would be scanned using PME, to take advantage of the ability to perform this procedure at the time of a primary care physician visit. Therefore, in these cohorts, it is assumed that for the patients who are scanned, aneurysms would be identified and treated prior to rupture.

The simulation model assumed that for each treatment scenario (e.g., Scenarios II - IV), patients were scanned in year 1 and then followed over a 5-year period without further scanning. Aneurysms were assumed to grow at rates consistent with that observed in the literature. Small aneurysm growth was assumed to follow a bi-modal path, with 50% growing at a rate of 0.27/cm/yr and the remaining 50% stable over time.[7, 11] Medium and large aneurysms were assumed to grow at a rate of 0.27/cm/yr.[11]

The simulation model is developed to quantify the expected costs and expected benefits in scanning and usual care treatment arms, respectively, for a hypothetical cohort of 10,000 patients. Costs and benefits are quantified as expected costs because of the inherent uncertainties associated with medical treatments. For example, surgical treatment for AAA patients is not 100% successful. Hence, benefits must be multiplied by the probability that the treatment in fact succeeds, yielding expected benefits; similar reasoning leads us to quantify expected costs. Model input values are taken from a variety of sources in the literature. The analysis is conducted from the third-party payer perspective.

### Calculating Expected Benefits

Benefits were measured by the number of quality-adjusted life years (QALYs) saved, which were estimated as the expected numbers of deaths in each treatment arm for each year of follow-up. In this model, deaths could occur from elective or emergency surgery, with a much higher mortality rate in the latter. These deaths were then multiplied by the life expectancy of those subjects had they not died. To calculate total QALYs saved, the life years saved were adjusted downward to reflect the actual quality of life (QoL) of subjects in this hypothetical study, which was assumed to be less than perfect health. In particular, QoL for subjects in perfect health is 1, whereas the subjects in the study cohorts were assumed to have a QoL of 0.70 on average.[7] Thus, life years saved from scanning were multiplied by 0.70 to arrive at the present value of QALYs saved. These QALYs were then summed and converted to present value terms using a 3% discount rate.

To illustrate, let $QALY_{uni}$=QALYs lost in each year for the unscanned treatment arm in year 1 and the period of follow-up is 5 years. Then the net present value of life years lost in the unscanned treatment arm, $PVQALY_{un}$, is calculated as:

(1) $PVQALY_{un}=QALY_{un1}+QALY_{un2}/(1+.03)+QALY_{un3}/(1+.03)^2+QALY_{un4}(1+.03)^3+QALY_{un5}(1+.03)^4$

Similar calculations yield the present value of QALYs lost for the other three treatment arms. Subtracting QALYs lost in one of the unscanned arms from those lost in the scanned arm provides the total number of QALYs saved from scanning relative to not scanning, ΔQALYSAVE. This will be a positive number because fewer life years are lost in the scanning treatment arms.

### Calculating Expected Costs

Costs included annual treatment costs in each arm as well as the costs of scanning in the scanning arms. Treatment costs included the costs of surgery and related hospitalization as well as costs due to any complications. These complications included dialysis-dependent renal failure, stroke, myocardial infarction, and major amputation. For each cohort, expected costs in each year were calculated. These costs were then expressed in present value terms, using a 3% discount rate.

To illustrate, denote annual costs in the unscanned treatment arm as $CST_{uni}$, and again assume a 5-year time horizon. Then the present value of treatment costs in the unscanned arm was calculated as:

(2) $PVCST_{un} =CST_{un1}+CST_{un2}/(1+.03)+CST_{un3}/(1+.03)^2 +CST_{un4}/(1+.03)^3+CST_{un5}/(1+.03)^4$

Similar calculations yielded the present value of treatment costs in the scanned treatment arms. Subtracting costs in each scanned arm from the unscanned arm provided the incremental cost of scanning relative to not scanning.

### Calculating Incremental Cost Utility Ratios

Once expected costs and benefits were calculated, incremental cost utility ratios were calculated. Using the subscript "sc" to denote a scanned cohort, the incremental cost utility ratio is expressed as:

(3) $\Delta PVCST/\Delta QALYSAVE = (PVCST_{sc} - PVCST_{un})/PVQALY_{un} - PVQALY_{sc})$

### Sensitivity Analysis

To gauge the robustness of the analysis to alternative plausible model input values, two sensitivity analyses were performed. The first method adjusted the model parameters simultaneously and randomly for 1,000 iterations of the model using in a Monte Carlo Simulation. This method provided estimates on the variability of the cost effectiveness expressed as a probability with respect to three threshold values, $50,000; $100,000; and dominance. The second method was a one-way sensitivity analysis. This method allowed for the identification of model parameters that had the greatest influence on the results. For both sensitivity analyses, cost and clinical endpoints were varied plus or minus 25% from their baseline values and the cost utility analysis recalculated in each analysis. The ranges chosen for the sensitivity analysis were determined to explore the robustness of the results over a wide range of scenarios.[12] The discount rate was also varied, from a low of 1% to a high of 5%.

## RESULTS

Table 1 shows the basic input values for the components of the model. As the table indicates, most AAAs are small (e.g., 4 cm). However, a significant proportion of these aneurysms (27%) are medium or large. These aneurysms are at much higher risk of rupture than are small aneurysms. While small aneurysms rupture at an average annual rate of 1%, rupture rates are 6% for medium aneurysms and 25% for large ones.

Table 1 also reveals that mortality rates from AAA surgery differ dramatically depending upon whether the surgery is performed on an elective (3%) or emergency (41%) basis. Emergency AAA surgery is also considerably more expensive ($116,493) than elective surgery ($73,904). The high rupture rates of medium and large AAAs and the high mortality and cost of emergency surgery all suggest that scanning to identify and treat AAAs electively may improve outcomes while constraining costs. Against this one must weigh the cost of scanning itself, which is $30 per scan, as shown in Table 1.

**Table 1.** Model Inputs and Values

| MODEL COMPONENT | Value | Source Ref# |
|---|---|---|
| ELIGIBLE POPULATION | | |
| Male Smokers aged 65+ | 10,000 | |
| SURVEILLANCE DISTRIBUTION | | |
| Scanned | 10,000 | Assumption |
| TOTAL AAA | 551 | [4] |
| Small (4.0-4.9 cm) | 402 | [25] |
| Medium (5.0-5.4 cm) | 83 | [25] |
| Large (≥5.5 cm) | 66 | [25] |
| ANEURYSM GROWTH & RUPTURE RATES | | |
| **Aneurysm growth rate (annual)** | | |
| Small | 50%: 0.27 cm; 50%: stable | |
| Medium | 0.27 cm | [7] |
| Large | 0.27 cm | [7] |
| **Aneurysm rupture rate (annual)** | | |
| Small (4.0 cm) | 1% | [25] |
| Medium (5.0 cm) | 6% | [25] |
| Large (5.5 cm) | 25% | [25] |
| MORTALITY RATE FROM SURGERY | | |
| Elective | 3% | [7]* |
| Emergency | 41% | [7] |
| COMPLICATION RATES FROM SURGERY | | |
| Dialysis-related renal failure | 0.5% elective; 3.8% emergency | [7, 26] |
| Stroke | 1.0% elective; 1.1% emergency | [7, 26] |
| Myocardial infarction | 2.9% elective; 2.9% emergency | [7, 26] |
| Major amputation | 0.1% elective; 0.5% emergency | [7, 26] |
| COSTS | | |
| Surgery | $73,904 elective; $116,493 emergency | [27] |
| **Complications (annual)** | | |
| Dialysis dependent renal failure | $99,030 annually | [7] |
| Stroke | $84,691 1st yr; $44,506 yrs 2-5 | [26] |
| Myocardial infarction | $18,213 1st yr; $4,636 yrs 2-5 | [26] |
| Major amputation | $102,242 1st yr; $65,791 yrs 2-5 | [26] |
| AAA surveillance w/ hand held ultrasound scan | $30 | [28] |
| QUALITY OF LIFE | 0.7 | [7] |
| AVERAGE LIFE EXPECTANCY | 13 yrs | [29]** |
| DISCOUNT RATE | 3% | [7, 26] |

AAA=abdominal aortic aneurysm;
*Young et.al compared two different surgical techniques. One had a mortality rate of 1.2 and the other technique had a mortality rate of 4.7. We used 3.0, the midpoint of this range;
**We assume that the average age of the 65+ male cohort is 71.

Table 2 shows the expected costs and QALYs lost under each scenario. In addition, Scenario I, which involved no scanning, had the most expected AAA-related deaths (71.8 for the cohort of 10,000). Scenario

II, which included scanning and surgical treatment for all identified aneurysms, had the fewest expected deaths, at 38.6. However, Scenario II also demonstrated the highest costs, at $42 million, resulting from the large number of AAA surgeries performed due to increased scanning. Compared to Scenario I, both Scenarios III and IV proved to cost less and had fewer expected deaths.

**Table 2.** Expected Total Costs and QALYs for Each Cohort

| | Scenario I | Scenario II (treat all) | Scenario III (treat medium & large) | Scenario IV (treat large) |
|---|---|---|---|---|
| TOTAL EXPECTED COSTS ($ MILLIONS) | $20.4 | $42.0 | $19.6 | $17.8 |
| QALYs Lost | 492 | 150 | 227 | 309 |

QALY=quality-adjusted life year

The results of the cost utility analysis are provided in Table 3 with the results of the probabilistic sensitivity analysis. Scenarios III and IV were dominant strategies to Scenario I; that is, they were less costly and involved fewer AAA-related deaths. The incremental cost utility ratio for Scenario II vs. Scenario I was $64,156 per quality-adjusted life year gained, which is below $100,000 threshold for the value of a QALY, suggesting that Scenario II is also cost effective relative to Scenario I.

**Table 3.** Incremental Costs/QALY for each Treatment Group Relative to Scenario I

| | Scenario II vs. Scenario I | Scenario III vs. Scenario I | Scenrio IV vs. Scenario I |
|---|---|---|---|
| COST/QALY | $64 156 | Scenario III is dominant* | Scenario IV is dominant* |
| Probability of Cost-Effectiveness at $50 000 Threshold | 0.25 | 1 | 1 |
| Probability of Cost-Effectiveness at $100 000 Threshold | 0.87 | 1 | 1 |

QALY=quality-adjusted life year;
*Scenario III saves 265 more QALYs and costs $800,000 less than Scenario I;
Scenario IV saves 183 more QALYs and costs $2.6 million less than Scenario I.

Next, we compare the cost effectiveness of scanning and treating all identified aneurysms (Scenario II) to Scenarios III and IV, respectively, which only treated a portion of aneurysms. This analysis revealed that Scenario II was not cost effective relative to Scenario III (surgery for medium or large AAA; incremental cost-effectiveness ratio (ICER) =$291,785) or Scenario IV (surgery for large AAA only; ICER =$151,924). Furthermore, ICERs were calculated comparing Scenarios III and IV in terms of cost effectiveness. The ICER for Scenario III relative to IV demonstrated that Scenario III costs were $21,704 higher per QALY gained.

To gauge the reliability of these results and understand which model parameters drove the results, one-way sensitivity analysis was performed, in addition to the probabilistic sensitivity analysis discussed earlier. The results of the sensitivity analyses are reported in Table 4. In all cases, the original conclusions were confirmed for Scenarios III and IV, which continued to dominate Scenario I. In the vast majority of cases, Scenario II remained cost effective with respect to Scenario I. The only exceptions occurred when aneurysm rupture rates and the probability of death during emergency surgery were assumed to be low. The results were most sensitive to variations in rupture rates, emergency surgery mortality, quality of life, and life expectancy.

**Table 4.** One-way Sensitivity Analysis: Incremental Cost/QALY Under Alternative Model Input Value

| MODEL INPUT | SCENARIO II VS SCENARIO I | SCENARIO III VS SCENARIO I | SCENARIO IV VS SCENARIO I |
|---|---|---|---|
| **PREVALENCE AAA** | | | |
| Hi | $63 980 | -$2008 | -$12 716 |
| Lo | $64 446 | -$1407 | -$11 844 |
| **PROBABILITY MORTALITY EMERGENCY SURGERY** | | | |
| Hi | $47 183 | -$1383 | -$9718 |
| Lo | $100 145 | -$2502 | -$17 074 |
| **PROBABILITY MORTALITY ELECTIVE SURGERY** | | | |
| Hi | $72 048 | -$1852 | -$12 700 |
| Lo | $57 822 | -$1716 | -$12 090 |
| **ANEURYSM RUPTURE RATE** | | | |
| Hi | $41 102 | -$7768 | -$16 229 |
| Lo | $111 305 | $9224 | -$5401 |
| **COMPLICATION RATE** | | | |
| Hi | $64 260 | -$2257 | -$12 954 |
| Lo | $64 055 | -$1310 | -$11 825 |
| **COST OF SURGERY** | | | |
| Hi | $79 871 | -$2036 | -$15 331 |
| Lo | $48 441 | -$1527 | -$9445 |
| **AAA SCANNING COSTS** | | | |
| Hi | $64 375 | -$1499 | -$11 978 |
| Lo | $63 937 | -$2064 | -$12 797 |
| **QUALITY OF LIFE** | | | |
| Hi | $51 325 | -$1425 | -$9910 |
| Lo | $85 541 | -$2375 | -$16 517 |
| **LIFE EXPECTANCY** | | | |
| Hi | $48 106 | -$1372 | -$9672 |
| Lo | $96 278 | -$2539 | -$17 223 |
| **DISCOUNT RATE** | | | |
| Hi | $57 479 | -$3381 | -$13 227 |
| Lo | $71 126 | -$179 | -$11 560 |

QALY=quality-adjusted life year; AAA=abdominal aortic aneurysm

## DISCUSSION

This study developed a cost effectiveness simulation model to investigate the economic feasibility of opportunistic scanning for AAA using a PME device in the primary care physician office. The results indicate that such scanning is cost effective, particularly when surgical intervention for AAA is limited to medium and/or large aneurysms. These aneurysms are at much greater risk of rupture than small aneurysms, so that surgical intervention is cost saving. Indeed, both the base case and sensitivity analysis indicated that use of the PME device to detect AAA is a dominant strategy when surgical intervention is confined to medium and/or large aneurysms.

While most of the results confirm that the PME device is also cost effective when all identified aneurysms are treated surgically, the results are far less compelling than for intervening on medium or large aneurysms only. Current guidelines recommend intervention for large aneurysms (≥5.5 cm AAA), while the decision to intervene surgically for aneurysms between 4.0 cm and 5.4 cm remains controversial.[2] Our findings suggest that surgical intervention to treat medium or large aneurysms only is more economically efficient.

To decide between Scenario III (treat medium and large) and Scenario IV (treat only large) the ICER was calculated ($21,704) and found to be well below the currently accepted threshold of $100,000 per QALY. A probabilistic sensitivity analysis found that Scenario III was cost-effective 99% of the time using a $100,000 threshold per QALY. One-way sensitivity analysis revealed the model was most sensitive to variations in rupture rates, and emergency surgery mortality. These findings suggest that treating medium as well as large aneurysms surgically is a cost effective alternative to treating only large aneurysms when scanning is performed using a PME in the office setting.

Earlier studies have examined the cost effectiveness of traditional larger ultrasound devices for AAA population scannings.[13-20] While a number of these studies suggest that such scanning is cost effective, others are less favorable.[21-23] A Cochrane systematic review of the cost effectiveness of AAA population scanning using traditional ultrasound devices concluded that while the cost effectiveness may be acceptable, further expert analysis was warranted to determine whether a coordinated population-based scanning program should be introduced.[21]

One study found quick ultrasound scans performed by vascular technologists (e.g., no more than 5 minutes) were cost effective in AAA population scanning.[24] However, that study was based on evidence from a small sample of just 25 patients and may not be generalizable to other patients and settings. The present study used clinical and economic data based on studies involving larger patient sample sizes in order to mitigate issues of robustness and generalizability. In contrast to previous studies using traditional ultrasound devices, this study showed that the PME device actually reduced costs when surgical intervention was restricted to patients with aneurysm at 5 cm or above, consistent with current guidelines. While the cost effectiveness of AAA scanning using traditional ultrasound devices remains uncertain,[6] the much lower cost of the PME device, the ability to be performed in a primary care physician's office, together with its high degree of accuracy, strengthen the case for AAA scanning.

Nevertheless, this study has some important limitations that must be noted. First, the results pertain to a population of older (average age of 71 years) male smokers and may not be generalizable to other populations. Second, the CUA was performed from the perspective of third-party payer and the results may differ when alternative perspectives are taken. Given the patient benefits under scenarios II-IV however, it seems quite likely that the results would be similar if a societal perspective were taken. Third, differences in efficacy of the scan as a result of a patient's body mass index (BMI) are not well understood. This model assumed the efficacy would remain constant regardless of BMI. Fourth, as with any economic simulation model, model input values are taken from various sources in the literature rather than from a consistent data source, introducing heterogeneity and uncertainty into the analysis. However, the sensitivity analyses suggest that the results are quite robust to alternative model input values. Finally, this study assumed the physician using the PME device had been trained on the proper use of the device.

## CONCLUSION

Under all of the treatment arms, use of the PME scanning device is cost effective relative to the un-scanned cohort. Treating patients with medium or large aneurysms or just those with large aneurysms, but not patients with small aneurysms, substantially enhances the cost effectiveness of using the scanning device. These results suggest that primary care physicians with proper training on the PME scanning device should consider this device as a cost effective method to opportunistically scan and manage AAA patients among older males who have a history of smoking.

## ACKNOWLEDGEMENTS

The authors gratefully acknowledge the contributions of Paige Levatino to the design of Figures 1 and 2.

### Conflict of Interest Declaration

This study was funded by GE Healthcare. The publication of study results was not contingent on the sponsor's approval or censorship of the manuscript. Denver Phiri is an employee of GE Healthcare, the study sponsor. Peter J. Mallow is an employee of $S^2$ Statistical Solutions, Inc., which is the paid consultant to GE Healthcare. John A. Rizzo is a paid consultant to $S^2$ Statistical Solutions, Inc.
